# Oleanolic Acid Modulates the Gut–Liver Axis to Alleviate High-Fat Diet-Induced Hepatic Lipid Deposition in Nile Tilapia (*Oreochromis niloticus*)

**DOI:** 10.3390/microorganisms14061247

**Published:** 2026-06-02

**Authors:** Kai Yu, Xuhong Yang, Ruijie Guo, Kai Huang, Jiagang Deng

**Affiliations:** 1 Guangxi Key Laboratory of Efficacy Study on Chinese Materia Medica, Guangxi University of Chinese Medicine, Nanning 530200, China; bioneer@foxmail.com; 2 College of Animal Science and Technology, Guangxi University, Nanning 530004, China; xuhongcap@163.com (X.Y.); 2118401008@st.gxu.edu.cn (R.G.); 3 Guangxi Key Laboratory of TCM Formulas Theory and Transformation for Damp Diseases, Guangxi University of Chinese Medicine, Nanning 530200, China; 4 Guangxi Collaborative Innovation Center of Study on Functional Ingredients of Agricultural Residues, Guangxi University of Chinese Medicine, Nanning 530200, China; 5 University Engineering Research Center of Reutilization of Traditional Chinese Medicine Resourcs, Nanning 530200, China

**Keywords:** oleanolic acid, *Oreochromis niloticus*, gut microbiota, gut–liver axis, high-fat diet, non-alcoholic fatty liver disease, short-chain fatty acids

## Abstract

This study examined the protective mechanisms of oleanolic acid (OA) against high-fat diet (HFD)-induced hepatic steatosis and intestinal dysbiosis in Nile tilapia. Fish were allocated to four groups: normal diet (ND), HFD, and OA-supplemented HFD (50 and 250 mg/kg). After 42 days, physiological, biochemical, and histological assessments demonstrated that OA markedly reduced hepatic lipid accumulation, mitochondrial injury, and intestinal shortening. Transcriptomic analysis revealed that OA alleviated lipid dysregulation by inhibiting de novo lipogenesis and promoting lipid trafficking and β-oxidation, effectively reversing HFD-induced changes in the PPAR, MAPK, mTOR, and autophagy-lysosome signaling pathways. 16S rRNA sequencing indicated that OA increased microbial alpha diversity, suppressing HFD-associated taxa (e.g., *Nordella*) while enriching beneficial genera such as *Clavibacter*, *Bosea*, and *Bdellovibrio*. Importantly, OA treatment restored HFD-induced depletion of intestinal butyric acid and suppressed hepatic pro-inflammatory cytokines (*tnf-α*, *il-1β*), while upregulating growth-related factors (*igf1*). Correlation analysis confirmed strong associations between microbial alterations (*Nordella* and *Phreatobacter*) and hepatic lipid metabolism and inflammatory gene expression. Overall, OA mitigates metabolic stress in Nile tilapia by reconfiguring the gut–liver axis, integrating microbial restoration with precise regulation of hepatic nutrient-sensing and inflammatory pathways, providing a potential therapeutic strategy for lipid metabolism disorders in aquaculture.

## 1. Introduction

The rapid expansion of the aquaculture industry has prioritized intensive production models, a shift that has inadvertently increased the prevalence of metabolic disorders in farmed species. Among these, non-alcoholic fatty liver disease (NAFLD) has emerged as a major challenge to aquaculture sustainability. To maximize the “protein-sparing effect” and improve feed efficiency, high-fat diets (HFDs) are commonly employed across the industry to reduce production costs and nitrogenous waste [1,2]. However, chronic exposure to excessive dietary lipids often exceeds the physiological capacity for lipid transport, biosynthesis, and β-oxidation. This metabolic imbalance leads to ectopic lipid accumulation and systemic dysfunction [3,4]. Evidence in tilapia demonstrates that high-fat intake initiates a detrimental cycle of hyperlipidemia, hepatic steatosis, and oxidative stress [5,6,7,8]. This disruption further triggers chronic inflammation [9], endoplasmic reticulum (ER) stress [10,11], and DNA damage, ultimately resulting in cellular apoptosis and impaired autophagy [12,13,14,15,16]. Collectively, these pathological changes compromise growth performance and increase susceptibility to disease [1]. Accordingly, identifying targeted interventions to counteract diet-induced NAFLD is critical for maintaining the health and productivity of farmed fish populations.

The gut microbiota, recognized as a key “metabolic organ,” plays an essential role in nutrient metabolism and immune regulation [17,18,19]. In fish, HFDs generally reduce microbial diversity and disrupt intestinal homeostasis, contributing to metabolic dysfunction [20]. Studies in various teleosts, including Atlantic salmon (*Salmo salar*), Nile tilapia (*Oreochromis niloticus*), and ricefield eel (*Monopterus albus*), have shown that HFDs significantly alter the composition and function of the intestinal microbiota [21,22,23,24]. Beyond community structure, these microbes influence lipid metabolism through lipase secretion, short-chain fatty acid (SCFA) production, and bile acid regulation, enabling communication with and regulation of distal organs via the gut–liver axis [19]. Understanding these interactions is therefore essential for improving fish health and developing functional aquafeeds.

Oleanolic acid (OA), a pentacyclic triterpenoid widely distributed in numerous plant species, exhibits diverse bioactivities, including anti-inflammatory, antioxidant, and hepatoprotective effects [25]. In mammals, OA has been reported to reduce fatty liver by regulating lipid synthesis and transport [26,27]. OA also modulates gut microbiota diversity, promotes the growth of probiotics, and alters the Firmicutes/Bacteroidetes (F/B) ratio, suggesting its potential role in regulating lipid metabolism via the gut–liver axis [26,28]. Previous studies found that OA activates the autophagy–lysosome pathway to reduce hepatic lipid accumulation in tilapia [29]. Nevertheless, the mechanisms by which OA regulates lipid metabolism and the gut microbiota in aquatic species remain poorly understood. Therefore, this study systematically evaluates the therapeutic effects of OA on HFD-induced fatty liver in Nile tilapia and investigates the underlying molecular pathways through integrated microbiota and transcriptomic analyses.

## 2. Materials and Methods

### 2.1. Experimental Diets

OA (purity ≥ 98.0%) was obtained from Chengdu Push Bio-Technology (Chengdu, China). Based on previous formulations [29], two basal diets were prepared: a normal diet (ND; 3.62% crude lipid) and an HFD (14.83% crude lipid). Two experimental diets were subsequently produced by supplementing the HFD with 50 mg/kg or 250 mg/kg OA, designated as the low-dose (OAL) and high-dose (OAH) groups, respectively.

### 2.2. Fish Management and Experimental Design

Genetically Improved Farmed Tilapia (GIFT, *O. niloticus*) fry were sourced from Guangxi Angui Aquaculture Co., Ltd. (Nanning, China). The feeding trial was conducted at Guangxi University in accordance with the guidelines of the Animal Care and Use Ethics Committee of Guangxi University (No: GXU-2022-255).

A total of 240 healthy tilapia of uniform size (initial weight: 1.66 ± 0.05 g) were acclimated for two weeks in 3 × 1 × 1 m concrete tanks. Following acclimation, fish were randomly assigned to the four treatment groups: ND, HFD, OAL, and OAH. Each group consisted of three replicates, with 20 fish per tank (1 × 1 × 1 m), resulting in a total of 12 tanks. During the 42-day trial, fish were hand-fed twice daily (09:00 and 18:00) at 5% of body weight. Water quality was maintained at 25–32 °C, pH 6.5–7.2, and dissolved oxygen > 5.0 mg/L. Residual feed and waste were siphoned daily, and approximately one-third of the water was replaced 2–3 times per week.

### 2.3. Sampling

At the end of the 42-day trial, fish were fasted for 24 h prior to sampling. Ten fish per replicate were randomly selected and deeply anesthetized with MS-222 (100 mg/L). Body weight and length were recorded, followed by rapid dissection on ice. Blood was collected from the caudal vein, allowed to clot at 4 °C for 2 h, and centrifuged (3500 rpm, 10 min, 4 °C) to obtain serum. The liver was weighed to calculate the hepatosomatic index (HSI).

To ensure consistency, liver tissues were excised from the same anatomical lobe and processed as follows: (1) one portion was fixed in 4% paraformaldehyde for histology; (2) another portion was cut into 1 mm^3^ cubes and fixed in 2.5% glutaraldehyde for transmission electron microscopy (TEM); and (3) the remaining tissue was snap-frozen in liquid nitrogen and stored at −80 °C for biochemical assays, transcriptomics, and qPCR. The intact intestine was removed for length measurement, with mid-gut segments fixed in 4% paraformaldehyde for histological analysis. Intestinal contents were collected using sterile slides, snap-frozen, and stored at −80 °C for 16S rRNA sequencing.

### 2.4. Histological Analysis

Liver and mid-gut samples fixed in 4% paraformaldehyde were dehydrated in a graded ethanol series, cleared in xylene, and embedded in paraffin. Serial sections (5 μm thick) were cut and stained with hematoxylin and eosin (H&E). Slides were mounted with neutral resin and examined under an Olympus BX53 optical microscope (Olympus, Tokyo, Japan). Images were captured using an Olympus DP74 digital camera (Olympus, Tokyo, Japan) (exposure time: 1/2439 s; resolution: 96 dpi; bit depth: 24) with 20× objectives to represent 200× magnification fields. Morphometric measurements, including hepatocyte vacuole area ratio, intestinal villus height, muscular layer thickness, and goblet cell counts, were quantified using Image-Pro Plus 6.0 software.

### 2.5. Oil Red O Staining

Liver tissues were embedded in OCT compound and sectioned into cryosections (8–10 μm). Sections were stained with Oil Red O working solution for 10 min in the dark, differentiated in isopropanol, and counterstained with hematoxylin to visualize nuclei. After mounting with glycerol gelatin, hepatic neutral lipid accumulation was observed and photographed under an Olympus BX53 optical microscope (Olympus, Tokyo, Japan) using the same imaging parameters as described above (Olympus DP74 camera; 200× magnification).

### 2.6. Transmission Electron Microscopy (TEM)

Fresh hepatic tissues (1 mm^3^) were fixed in 2.5% glutaraldehyde at 4 °C overnight. Samples were post-fixed in osmium tetroxide, dehydrated through a graded acetone series, and embedded in epoxy resin. Ultrathin sections (60–80 nm) were cut and double-stained with uranyl acetate and lead citrate. Hepatic ultrastructure, including mitochondrial morphology, lipid droplet distribution, and autolysosome formation, was examined using a HITACHI HT7700 transmission electron microscope (Hitachi, Tokyo, Japan) at an accelerating voltage of 80 kV and a magnification of 5000×. Images were captured with a 3296 × 2472 pixel digital camera.

### 2.7. Transcriptome Sequencing

Total RNA was extracted from liver tissues using TRIzol reagent (Takara, Dalian, China). RNA integrity was verified by agarose gel electrophoresis, and purity was confirmed with a NanoDrop spectrophotometer (Thermo Fisher Scientific, Wilmington, DE, USA) (OD_260/280_ 1.8–2.2). RNA concentration was measured using a Qubit^®^ 2.0 Fluorometer (Thermo Fisher Scientific, Waltham, MA, USA). High-quality RNA samples were used for library preparation with the NEBNext^®^ Ultra^TM^ RNA Library Prep Kit (NEB, Ipswich, MA, USA) and sequenced on the Illumina NovaSeq 6000 platform. Raw reads were filtered using fastp v0.23.2 and aligned to the Nile tilapia reference genome (*Oreochromis_niloticus*_Ensembl_105) with HISAT2. Gene expression was quantified using featureCounts v2.0.0 and normalized as Fragments Per Kilobase of transcript per Million mapped reads (FPKM). Differentially expressed genes (DEGs) were identified using DESeq2 with thresholds of |log_2_FoldChange| > 1 and *p*-adjust < 0.05. Functional enrichment analyses were conducted using Gene Ontology (GO) and Kyoto Encyclopedia of Genes and Genomes (KEGG). A protein–protein interaction (PPI) network was constructed via the STRING database.

### 2.8. 16S rRNA Sequencing

Total genomic DNA was extracted from intestinal contents using the CTAB method. DNA purity and integrity were verified by agarose gel electrophoresis, and concentrations were measured with a Qubit^®^ 2.0 Fluorometer. The V3–V4 hypervariable region of the 16S rRNA gene was amplified using barcoded primers (515F: 5′-GTGYCAGCMGCCGCGGTAA-3′; 806R: 5′-GGACTACHVGGGTWTCTAAT-3′). PCR was performed in triplicate using Phusion^®^ High-Fidelity PCR Master Mix (NEB, Ipswich, MA, USA). Amplicons were pooled, purified, and used for library construction with the NEB Next^®^ Ultra DNA Library Prep Kit. Following quality assessment via Qubit and qPCR, sequencing was performed on the Illumina NovaSeq 6000 platform (PE250). Raw reads were filtered using fastp to obtain clean tags. Sequence denoising and amplicon sequence variant (ASV) generation were conducted using the DADA2 plugin in QIIME2. Taxonomic assignment was performed using the Silva 138.1 database (0.8 confidence threshold), and phylogenetic relationships were inferred via MAFFT. All samples were normalized prior to downstream analyses.

### 2.9. Short-Chain Fatty Acids (SCFAs) Analysis

Intestinal SCFA concentrations were determined using GC-MS/MS. Briefly, 20 mg of homogenized intestinal content was extracted with 1 mL of 0.5% (*v*/*v*) phosphoric acid. Following grinding and 10 min of vortexing, samples were ultrasonicated for 5 min and centrifuged (12,000× *g*, 10 min, 4 °C). A 100 μL aliquot of the supernatant was mixed with 500 μL of methyl tert-butyl ether (MTBE) containing an internal standard. After further vortexing and ultrasonication, the organic phase was collected for analysis.

Analysis was conducted on an Agilent 7890B GC system (Agilent, Santa Clara, CA, USA) coupled with a 7000D mass spectrometer (Agilent, Santa Clara, CA, USA), equipped with a DB-FFAP column (30 m × 0.25 mm × 0.25 μm). Helium was used as the carrier gas at a flow rate of 1.2 mL/min. A 1 μL sample was injected with a 5:1 split ratio, and the oven temperature was programmed from 50 °C (1 min) to 220 °C at 18 °C/min, held for 5 min. Injector and transfer line temperatures were set at 250 °C and 230 °C, respectively. Quantification was performed in multiple reaction monitoring (MRM) mode using standard calibration curves. All samples were analyzed in duplicate.

### 2.10. Quantitative PCR (qPCR)

Total RNA was reverse-transcribed into cDNA using the HiScript II Q RT SuperMix kit (Vazyme, Nanjing, China). qPCR was performed on a Bio-Rad CFX96 real-time PCR detection system with ChamQ Universal SYBR qPCR Master Mix. Relative gene expression was calculated using the 2^–ΔΔCT^ method, with *gapdh* serving as the internal control. Primer sequences are listed in Table S1.

### 2.11. Statistical Analysis

Data analysis was conducted using SPSS 26.0 and expressed as mean ± standard deviation (SD). Significant differences among groups were assessed by one-way ANOVA followed by Tukey’s post hoc test, whereas pairwise comparisons were performed using Student’s *t*-test. Spearman’s rank correlation was applied to evaluate relationships between microbial abundance and hepatic gene expression in R. Interaction networks were visualized with Cytoscape (v3.10.1). Statistical significance was defined as *p* < 0.05.

## 3. Results

### 3.1. OA Attenuates HFD-Induced Hepatic Lipid Accumulation and Intestinal Shortening

**Figure 1:**
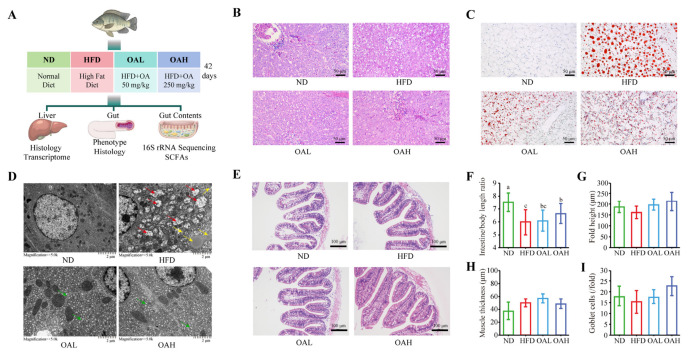
Effects of OA on HFD-induced hepatic and intestinal phenotypes. (**A**) Schematic of the experimental design. (**B**) Representative hepatic H&E staining images. (**C**) Representative hepatic Oil Red O staining images. (**D**) Hepatic ultrastructure via TEM (×5.0k); red arrows: swollen mitochondria; yellow arrows: lipid droplets; green arrows: dilated, vesicle-like RER. (**E**) Representative intestinal H&E staining images. (**F**–**I**) Statistical analysis of intestine/body length ratio (**F**) and histological parameters: villus height (**G**), muscular layer thickness (**H**), and goblet cell counts (**I**). Values are expressed as mean ± SD. Different letters indicate significant differences (*p* < 0.05).

The experimental workflow is depicted in Figure 1A. H&E staining demonstrated that hepatocytes in the ND group retained intact cellular architecture, with tightly packed cells and centrally located nuclei, exhibiting only minimal lipid vacuolization. In contrast, the HFD group displayed disorganized cellular cords, displaced nuclei, and extensive lipid vacuole accumulation. OA supplementation (OAL and OAH) effectively restored hepatocyte structure and reduced lipid droplet presence (Figure 1B). These observations were confirmed by Oil Red O staining, which indicated that OA treatment markedly alleviated HFD-induced hepatic lipid deposition (Figure 1C). TEM analysis further revealed alterations in hepatic ultrastructure (Figure 1D). Hepatocytes in the ND group exhibited normal morphology, with intact mitochondria and organelles. The HFD group, however, showed mitochondrial swelling, extensive lipid droplet accumulation, and general structural disintegration. OA treatment reduced lipid droplets, improved mitochondrial integrity, and induced expansion of the rough ER (RER) into vesicle-like structures, suggesting an adaptive ultrastructural reorganization to mitigate ER stress.

Concerning intestinal morphology (Figure 1E–I), HFD significantly decreased the intestine-to-body length ratio in tilapia, an effect reversed by OAH supplementation (Figure 1F). Histological analysis revealed no significant differences among groups in villus height, muscular layer thickness, or goblet cell counts (Figure 1G–I). These results indicate that OA attenuates HFD-induced hepatic steatosis and ultrastructural damage in Nile tilapia.

### 3.2. Liver Transcriptomic Profiles and GO Enrichment

**Figure 2:**
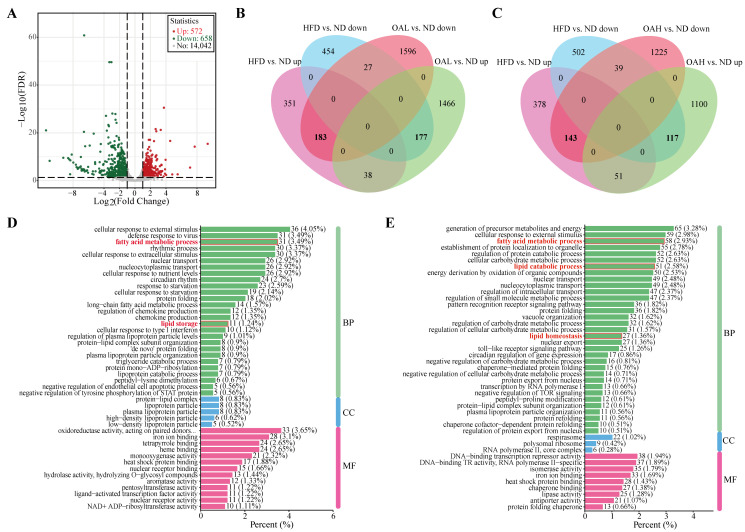
Hepatic transcriptomic profiles and GO enrichment analysis. (**A**) Volcano plot of DEGs in HFD vs. ND comparison. (**B**,**C**) Venn diagrams showing overlap of DEGs in OAL vs. HFD (**B**) and OAH vs. HFD (**C**). (**D**,**E**) GO enrichment analysis of reversed DEGs in OAL vs. HFD (**D**) and OAH vs. HFD (**E**).

The volcano plot (Figure 2A) illustrates significant alterations in hepatic gene expression in the HFD group relative to the ND group. A total of 1230 DEGs were identified, comprising 572 upregulated and 658 downregulated genes. Venn diagrams were employed to determine DEGs reversed by OA supplementation (Figure 2B,C). In the OAL group, 360 HFD-induced DEGs were reversed, including 177 restored and 183 suppressed genes. Similarly, OAH treatment reversed 260 DEGs, consisting of 117 restored and 143 suppressed genes. GO enrichment analysis was conducted to explore the biological functions of these reversed DEGs. In the OAL vs. HFD comparison, DEGs were significantly enriched in biological processes related to fatty acid metabolism (31 genes) and lipid storage (11 genes) (Figure 2D). For the OAH vs. HFD group, DEGs were predominantly associated with fatty acid metabolism (58 genes), lipid catabolism (51 genes), and lipid homeostasis (27 genes) (Figure 2E). Molecular function enrichment indicated strong links with oxidoreductase and binding activities. These results suggest that OA mitigates HFD-induced metabolic stress through the regulation of lipid metabolism and redox homeostasis. DEGs related to lipid metabolism are detailed in Table S2.

### 3.3. Integrative Protein–Protein Interaction (PPI) Network Analysis of Key KEGG Pathways

**Figure 3:**
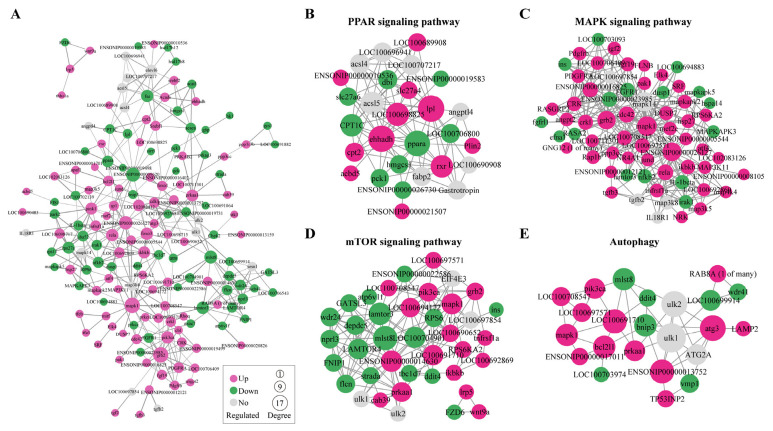
Interaction network of DEGs within key metabolic pathways. (**A**) Integrated PPI network illustrating cross-talk among the PPAR, MAPK, mTOR, and autophagy–lysosome pathways (confidence score > 0.7). (**B**–**E**) Pathway-specific sub-networks for PPAR (**B**), MAPK (**C**), mTOR (**D**), and autophagy (**E**) (confidence score > 0.4).

To examine functional interactions among the identified DEGs, a PPI network was constructed based on the top-enriched KEGG pathways. The integrated network revealed extensive connectivity and cross-talk across multiple metabolic and signaling modules, notably the PPAR, MAPK, mTOR, insulin signaling, and autophagy–lysosome pathways (Figure 3A). Within the PPAR signaling pathway, OA treatment markedly upregulated key metabolic regulators, including *ehhadh*, *loc100698825*, and *rxr*, while downregulating *ppara*, *lpl*, and *cpt1c* (Figure 3B), indicating a fine-tuned modulation of lipid oxidation and transport. Concurrently, the MAPK signaling pathway displayed strong activation, with significant upregulation of multiple *mapk*, *mapkk*, and *mapkkk* genes (Figure 3C). Moreover, OA treatment suppressed mTOR signaling through downregulation of several mTOR complex components, consistent with the induction of autophagy markers such as *atg3* and *gabarapl2* (Figure 3D). Broad induction of genes within the autophagy–lysosome pathway suggests that OA enhances lysosomal function and promotes metabolic flux (Figure 3E). Detailed information on these core DEGs and their fold changes is provided in Table 1.

### 3.4. qPCR Analysis of Key DEGs Derived from Transcriptome

**Figure 4:**
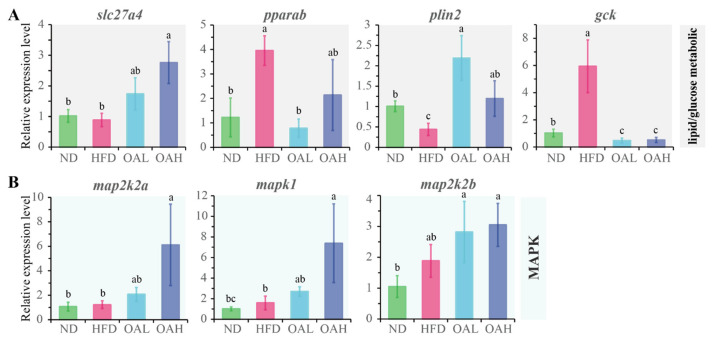
qPCR validation of key DEGs. (**A**) Relative expression of genes associated with lipid and glucose metabolism. (**B**) Relative expression of MAPK signaling pathway genes. Values are expressed as mean ± SD. Different letters indicate significant differences (*p* < 0.05).

To validate the transcriptomic findings, representative genes involved in lipid metabolism and signaling were selected for qPCR analysis. The results demonstrated that OA suppressed the expression of genes related to fatty acid transport (*slc27a4*), lipid droplet coating (*plin2*), and glycolysis (*gck*) (Figure 4A). Furthermore, OAH treatment significantly upregulated key components of the MAPK signaling cascade, including *map2k2a*, *map2k2b*, and *mapk1* (Figure 4B). These qPCR results were consistent with the transcriptomic trends, confirming the reliability of the sequencing data.

### 3.5. Effects of OA on the Intestinal Microbial Community in Nile Tilapia

**Figure 5:**
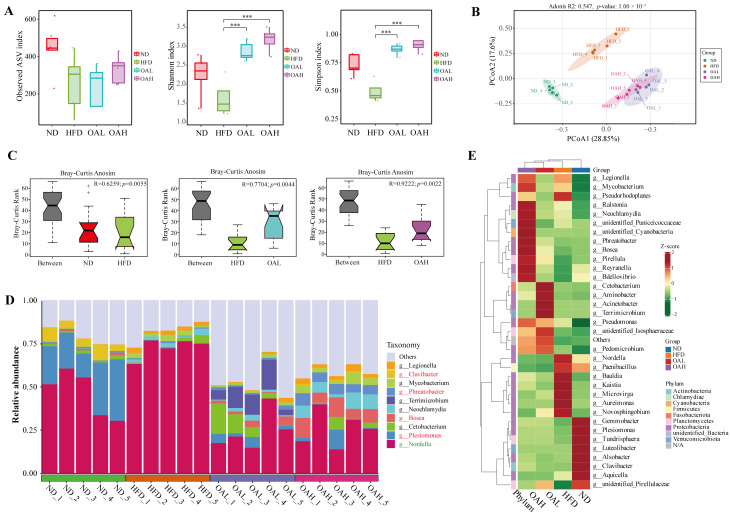
Intestinal microbial diversity and community composition. (**A**) Alpha diversity indices, including observed ASVs, Shannon, and Simpson indices. (**B**) Principal coordinates analysis (PCoA) based on unweighted UniFrac distances. (**C**) Anosim analysis of beta diversity based on unweighted UniFrac distances for ND vs. HFD, HFD vs. OAL, and HFD vs. OAH. (**D**) Relative abundance of microbiota at the genus level. (**E**) Cluster heatmap of dominant genera across groups. Data are presented as mean ± SD. *** *p* < 0.001.

Gut microbial profiling revealed that HFD significantly decreased alpha diversity, as indicated by Shannon and Simpson indices, relative to the ND group. OA supplementation (OAL and OAH) reversed this decline, restoring microbial diversity (Figure 5A). Beta diversity analysis using Principal Coordinates Analysis (PCoA) showed distinct clustering of microbial communities among groups, with low intra-group variation. Notably, the OAL and OAH groups exhibited similar community structures (Figure 5B). Anosim analysis based on Bray–Curtis distances confirmed significant differences between the ND and HFD groups, as well as between the HFD and OA-treated groups, with inter-group variation significantly exceeding intra-group variation (Figure 5C).

At the genus level, HFD feeding significantly altered the abundance of 13 genera, with 5 upregulated and 8 downregulated (Table S3). Among the top 10 genera, *Nordella* was the predominant taxon across all groups. HFD increased the relative abundances of *Nordella*, *Mycobacterium*, *Legionella*, and *Neochlamydia*, while decreasing *Bosea*, *Plesiomonas*, and *Clavibacter*. OA supplementation notably reduced *Nordella* abundance and elevated the proportions of *Clavibacter*, *Phreatobacter*, and *Bosea* (Figure 5D). Additionally, OA treatment restored the abundance of several other genera, including unidentified *Pirellulaceae*, *Plesiomonas*, *Bdellovibrio*, *Gemmobacter*, and *Kaistia*, to levels comparable to the ND group, as further confirmed by the cluster heatmap (Figure 5E).

### 3.6. Analysis of Predominant Taxa and Functional Profiling

**Figure 6:**
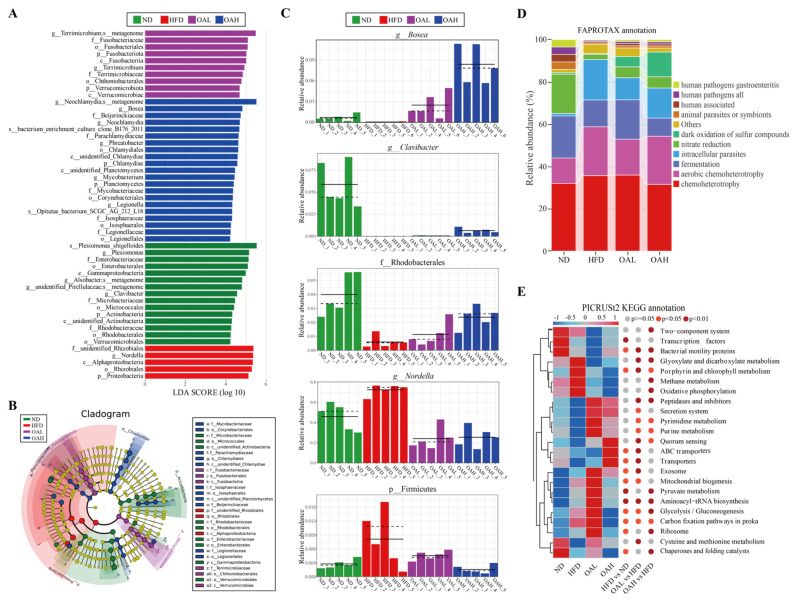
Identification of microbial biomarkers and functional prediction. (**A**) LDA scores from LEfSe analysis. (**B**) ASV-based cladogram representing taxonomic hierarchy of biomarkers. (**C**) Relative abundance of representative differential taxa. (**D**) FAPROTAX-based functional profiling. (**E**) Heatmap of KEGG metabolic pathways predicted by PICRUSt2.

Linear discriminant analysis effect size (LEfSe) identified 44 differentially enriched microbial biomarkers across the four groups, with 12 in ND, 4 in HFD, 6 in OAL, and 22 in OAH (Figure 6A). The taxonomic hierarchy of these biomarkers was visualized in an ASV-based cladogram (Figure 6B). Notably, OA supplementation enriched *Bosea*, *Clavibacter*, and *Rhodobacteraceae*, while effectively reversing the HFD-induced accumulation of *Nordella* and Firmicutes (Figure 6C).

FAPROTAX-based functional profiling revealed that HFD upregulated pathways related to chemoheterotrophy, aerobic chemoheterotrophy, and intracellular parasites, whereas pathways associated with nitrate reduction and human pathogens were downregulated (Figure 6D). OA supplementation (OAL and OAH) reversed these functional alterations, indicating a restorative effect on microbial metabolic stability. Furthermore, PICRUSt2-based KEGG pathway analysis demonstrated that OA significantly enhanced purine metabolism, pyrimidine metabolism, and quorum-sensing pathways relative to the HFD group. Conversely, pathways including glyoxylate and dicarboxylate metabolism, methane metabolism, and oxidative phosphorylation were suppressed (Figure 6E). These results suggest that OA modulates host energy homeostasis by reshaping the gut microbial metabolic network.

### 3.7. Correlation Between the Gut Microbiota and Hepatic Gene Expression

**Figure 7:**
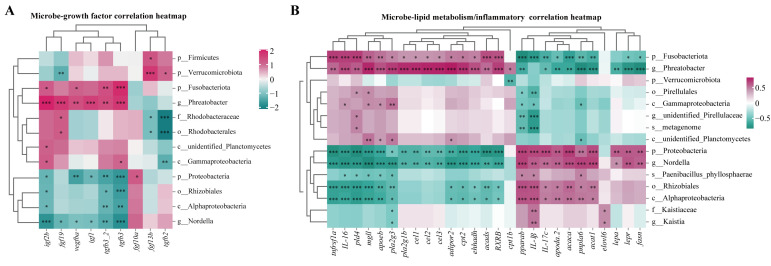
Correlation between intestinal microbiota and hepatic gene expression. (**A**) Spearman correlation between growth factor expression and microbial abundance. (**B**) Spearman correlation between lipid metabolism/inflammation-related genes and microbial abundance. Red and blue indicate positive and negative correlations, respectively. * *p* < 0.05, ** *p* < 0.01, *** *p* < 0.001.

Spearman’s correlation analysis was performed to explore relationships between the altered gut microbiota and host hepatic physiological responses. Specific taxa exhibited distinct associations with growth factors and cytokines (Figure 7A). The abundances of Fusobacteriota/*Phreatobacter* were positively correlated with growth-related genes, including *igf2b*, *fgf19*, *vegfba*, *igf1*, and *tgfb*. In contrast, Proteobacteria members (e.g., *Nordella*, Rhizobiales, and Alphaproteobacteria) displayed negative correlations with these growth factors. Figure 7B illustrates correlations between microbiota and genes involved in lipid metabolism and inflammation. *Phreatobacter* positively correlated with pro-inflammatory markers (*tnfrsf1a*, *il-16*, and *pld4*) and lipid metabolism-related genes (*cpt2*, *ehhadh*, *acads*, *rxrb*, and *cels*), while exhibiting negative correlations with *pparab*, *il-1b*, *il-17c*, and *acaca*. Proteobacteria demonstrated an approximately opposite correlation pattern. These results indicate that the enrichment of *Phreatobacter* and depletion of *Nordella* may play key roles in regulating growth signaling, lipid metabolic flux, and inflammatory responses. These results highlight the potential of targeting the gut microbiota to manage metabolic disorders in farmed fish.

### 3.8. OA Modulates the Expression of Hepatic Cytokines

**Figure 8:**
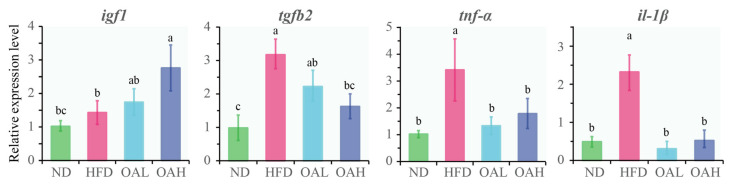
qPCR analysis of hepatic cytokines. mRNA expression levels of liver inflammation- and growth-related genes. Values are presented as mean ± SD. Different letters indicate significant differences (*p* < 0.05).

Hepatic cytokine mRNA levels were quantified via qPCR. Relative to the ND group, fish fed an HFD exhibited significantly elevated transcript levels of pro-inflammatory cytokines, including *tgfb2*, *tnf-α*, and *il-1β*, accompanied by an upward trend in *igf1* expression. These findings indicate that HFD consumption elicits a pronounced hepatic inflammatory response and modulates the expression of growth-related genes. Compared to the HFD group, OAH supplementation significantly downregulated *tgfb2*, *tnf-α*, and *il-1β* mRNA levels, while upregulating *igf1*. In contrast, OAL treatment effectively suppressed tnf-α and *il-1β* expression, although its effects on *igf1* and *tgfb2* did not reach statistical significance (Figure 8).

### 3.9. Effects of OA on Intestinal SCFAs Profiles

**Figure 9:**
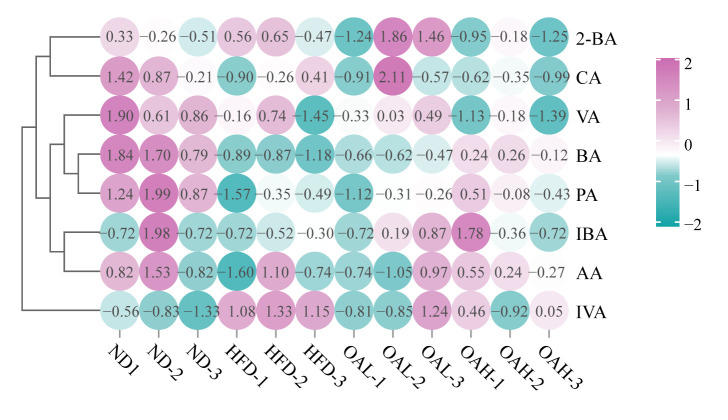
Intestinal SCFA profiles. Concentrations of SCFAs in intestinal contents across different treatment groups.

Hierarchical clustering of intestinal SCFAs revealed distinct metabolic profiles among the experimental groups. Notably, concentrations of butyric acid (BA) and isovaleric acid (IVA) were significantly altered in OAH relative to HFD (Figure 9). HFD feeding markedly reduced BA levels compared to the ND group, a trend partially restored by OA supplementation, suggesting that OAH may support gut energy metabolism and epithelial homeostasis by modulating butyrate-producing microbiota. Additionally, the observed shifts in IVA concentrations reflect alterations in metabolic pathways associated with branched-chain amino acid fermentation.

## 4. Discussion

### 4.1. OA Ameliorates HFD-Induced Fatty Liver by Modulating Lipid Metabolism

#### 4.1.1. Fatty Acid Synthesis

Endogenous fatty acid synthesis is predominantly regulated by fatty acid synthase (*fasn*) and acetyl-CoA carboxylase (*acc*), the rate-limiting enzymes in de novo lipogenesis. Although high-fat intake generally induces the upregulation of these enzymes in mammals [30] and medaka [31], *fasn* was significantly downregulated in HFD-fed tilapia (Table S2). This observation aligns with Rui et al. [11], who also reported a reduction in *acc* expression, whereas acc remained stable in the present study. Such differences may reflect variations in diet composition or adaptive metabolic states. Notably, OA treatment markedly suppressed both *fasn* and *acc1* (Table S2), indicating a robust inhibitory effect on de novo lipogenesis. Beyond the canonical cytoplasmic pathway, OA downregulated *hsd17b8* (Table S2), a gene involved in mitochondrial fatty acid synthesis often implicated in NAFLD progression [32,33]. Additionally, OA reduced the expression of acyl-CoA thioesterases (*acots*), key mediators of fatty acid metabolism. These results suggest that OA alleviates hepatic lipid accumulation by broadly inhibiting multiple fatty acid biosynthetic pathways.

#### 4.1.2. Lipid Transport

Lipoprotein lipase (LPL) serves as the primary rate-limiting enzyme for plasma triglyceride (TG) clearance, catalyzing the hydrolysis of TGs in chylomicrons and very-low-density lipoproteins (VLDL) [34,35]. In this study, HFD feeding downregulated *lpl* and *ENSONIG00000002328*. OA intervention produced differential effects: *lpl* remained suppressed, whereas *ENSONIG00000002328* was significantly upregulated (Table S2), suggesting that *ENSONIG00000002328* may act as the predominant functional ortholog mediating lipid hydrolysis in Nile tilapia under these conditions. Furthermore, OA appeared to modulate the systemic apolipoprotein network, evidenced by the upregulation of key apolipoproteins, including *apoa4a* (Apo-AIV), *apoeb* (Apo-E), and *apob* orthologs (*ENSONIG00000014666*, *ENSONIG00000040595*) (Table S2). Simultaneously, the LPL inhibitor *apoc1* (*ENSONIG00000027502*) was significantly downregulated [36]. These coordinated changes suggest that OA may enhance LPL catalytic efficiency and optimize lipid transport and redistribution by reshaping the apolipoprotein profile, thereby mitigating ectopic lipid deposition and potentially restoring insulin sensitivity in HFD-fed fish.

#### 4.1.3. Fatty Acid Oxidation

Deficient hepatic fatty acid oxidation is a central contributor to NAFLD. Mitochondrial β-oxidation initiates with the conversion of long-chain fatty acids into acyl-CoA by long-chain acyl-CoA synthetases (ACSL) [37]. HFD feeding suppressed *acsl5*, *acsl3b*, and *acsl4a* expression, whereas OA did not restore these specific isoforms but strongly upregulated another family member, *ENSONIG00000008380*, indicating a potential compensatory response. In contrast, short-chain acyl-CoA synthetases (acss) generally decreased, except for *acss2*, which was upregulated by high-dose OA (Table S2). ACSS enzymes convert SCFAs (e.g., acetate) into acetyl-CoA, linking carbon metabolism to cholesterol synthesis [38].

For mitochondrial fatty acid transport, the CPT1–CACT–CPT2 system mediates acyl group translocation across membranes. OA significantly increased *cpt1b* and *cpt2* transcript levels while bidirectionally regulating *cact* (*crata*/*cratb*). Additionally, OA enhanced the expression of *ehhadh* and *auh* (Table S2), two pivotal enzymes in the β-oxidation pathway. These results indicate that OA likely facilitates hepatic lipid clearance by strengthening the molecular machinery for β-oxidation.

#### 4.1.4. Cholesterol and Carbohydrate Metabolism

Disrupted cholesterol homeostasis represents a defining feature of NAFLD. Lysosomal acid lipase (LAL, encoded by *lipa*) preserves hepatic lipid equilibrium by hydrolyzing cholesteryl esters and TG [39,40]. In the present context, HFD feeding induced upregulation of carboxyl ester lipase (*cel*) genes, with OA treatment further enhancing this response, potentially reflecting increased lysosomal cholesterol degradation. Concurrently, OA modulated the expression of *soat1* and *soat2* while suppressing key cholesterol biosynthetic genes, including *fdft1*, *lss*, *dhcr24*, *ebp*, and *ebpl* (Table S2), indicating an inhibitory effect on both cholesterol esterification and de novo synthesis [41].

In relation to carbohydrate metabolism, glucokinase (*gck*) plays a critical role in hepatic glucose utilization [42]. Nevertheless, sustained *gck* overexpression under HFD conditions can promote lipogenesis and lipid deposition [43]. OA treatment reversed HFD-induced *gck* upregulation (Table S2), suggesting mitigation of lipid accumulation through restriction of metabolic flux from glucose to lipid synthesis, thereby supporting metabolic homeostasis.

Collectively, OA orchestrates a comprehensive reprogramming of the hepatic lipid metabolic network. By suppressing de novo fatty acid and cholesterol synthesis, enhancing lipid transport and β-oxidation, and limiting glucose-to-lipid conversion, OA acts synergistically to alleviate HFD-induced metabolic disturbances in Nile tilapia.

### 4.2. OA-Mediated Regulation of the Gut Microbiota in Nile Tilapia

#### 4.2.1. OA Reshapes the Structure and Composition of the Gut Microbiota

Beyond its direct influence on hepatic lipid metabolism, OA functions as a potent modulator of the gut microbiota, which represents a critical interface for host metabolic homeostasis [44]. Given that NAFLD pathogenesis is closely associated with gut dysbiosis, restoration of microecological balance constitutes a key therapeutic strategy. In the present study, HFD feeding reduced microbial diversity, whereas OA supplementation markedly increased community richness and evenness, as reflected by elevated Shannon and Simpson indices (*p* < 0.001, Figure 5A). Moreover, Bray–Curtis dissimilarity analysis demonstrated that OA substantially reshaped the microbial landscape (R > 0.77, *p* < 0.01, Figure 5C), indicating a pronounced shift in community composition.

At the genus level, HFD induced expansion of pathogenic taxa, notably *Mycobacterium* and *Legionella*, while suppressing potential probiotics such as *Bdellovibrio* (Figure 5D,E). OA treatment effectively reversed these alterations. *Mycobacterium* is a well-known aquaculture pathogen capable of causing systemic infections and severe economic losses in tilapia farming [45,46]. Similarly, *Legionella* is an opportunistic pathogen that typically declines following probiotic administration [47]. Conversely, *Bdellovibrio*, a predatory prokaryote, acts as a promising biocontrol agent due to its antimicrobial activity against *Vibrio* and *Salmonella* [48,49]. OA also restored the abundance of *Rhodobacterales*, a group notable for metabolic versatility and roles in nutrient cycling and pathogen suppression [50,51,52]. These taxonomic shifts indicate that OA promotes a healthier intestinal environment by suppressing opportunistic pathogens and enhancing populations of beneficial, metabolically diverse bacteria.

At the phylum level, OA significantly enriched Fusobacteriota, consistent with prior reports showing that probiotics or fermentation products elevate Fusobacteriota abundance, thereby improving hepatic health and intestinal integrity in HFD models [53,54]. The proliferation of such beneficial taxa can stabilize the gut ecosystem and mitigate metabolic inflammation, suggesting that OA alleviates HFD-induced dysbiosis by restoring these symbiotic populations. Additionally, a notable increase in Planctomycetes was observed in OA-treated groups. As common teleost commensals, Planctomycetes utilize carbohydrate fermentation under anaerobic conditions [55,56]. Their pronounced response to OA suggests that this triterpenoid may also modulate the intestinal carbohydrate landscape, further contributing to microbial homeostasis.

#### 4.2.2. OA-Driven Modulation of Gut Microbial Functional Profiles

The intestinal NO_3_^−^/NO_2_^−^/NO pathway plays a central role in host physiology by converting dietary nitrate into bioavailable NO and other reactive nitrogen species [57]. This pathway contributes to the resolution of intestinal inflammation and the regulation of hepatic lipid homeostasis, thereby providing protection against NAFLD progression [58]. High-nitrate interventions have been shown to activate this axis, ameliorating HFD-induced metabolic syndrome and hepatic steatosis through AMPK activation and suppression of oxidative stress [59,60,61]. In the present study, FAPROTAX-based functional prediction indicated that OA treatment partially reversed the HFD-induced depletion of nitrate-reducing bacteria (Figure 6E), suggesting that OA may exert hepatoprotective effects by potentially enhancing microbial-derived NO production.

### 4.3. Potential Mechanisms of the Regulation of Gut–Liver Axis by OA

Bile acids serve as key signaling molecules within the enterohepatic circulation, and their metabolic pool is tightly regulated by the gut microbiota [62,63]. Carboxyl ester lipase (CEL, encoded by *cel*), a critical enzyme for hydrolyzing cholesteryl esters and TG, directly impacts hepatic lipid mobilization. Although bile acid composition was not profiled in this study, transcriptomic analysis revealed that OA significantly upregulated *cel* expression. Spearman correlation analysis showed that cel levels were negatively associated with *Nordella* (*p* < 0.01) and positively associated with *Phreatobacter* (*p* < 0.001) (Figure 7), indicating that OA may facilitate microbial–host communication by modulating bile salt metabolism. Commensal microbes can alter the bile acid pool via bile salt hydrolase activity, subsequently influencing farnesoid X receptor (FXR) signaling, a master regulator of lipid metabolism [64,65]. These data suggest that OA orchestrates the gut–liver axis by shifting the abundance of taxa such as *Nordella* and *Phreatobacter*, potentially affecting the bile acid–FXR cascade to regulate metabolic genes, including *cel*.

Correlation analysis further revealed a strong metabolic coupling between the gut microbiota and hepatic transcription. Notably, *Nordella* and *Phreatobacter* displayed opposing correlation patterns with lipid-metabolic and inflammatory markers, suggesting an antagonistic interplay between these genera (Figure 7). While *Nordella* remains poorly characterized [66], emerging evidence highlights the biological relevance of *Phreatobacter*. In broilers, Phreatobacter abundance correlates with splenic *il-2* and *ifn-γ* expression [67]. In aquaculture, dietary composition or glucose load markedly affects Phreatobacter populations in grass carp, which are associated with endothelial *ctsc* expression [68,69]. These findings link *Phreatobacter* to immune pathways and intestinal integrity. Berberine intervention aimed at mitigating lipid disorders in grass carp also enriched *Phreatobacter* [70]. Together with the present results, these findings support a role for *Phreatobacter* as a microbial mediator of lipid metabolism and energy homeostasis in teleosts.

Microbial-derived SCFAs, particularly butyrate, act as critical signaling molecules coordinating host energy balance. Elevating intestinal butyrate has been shown to mitigate HFD-induced hepatic steatosis by restructuring the microbial community and activating SCFA-sensitive receptors [71]. For example, in largemouth bass, higher butyrate concentrations correlate with downregulation of lipogenic genes (e.g., *srebp1*, *fasn*) and upregulation of catabolic markers (e.g., *ppara*, *cpt1*) [72]. Mechanistically, butyrate promotes fatty acid β-oxidation via the *ppara*/*cpt1a*/*acaa2* axis while suppressing SREBP-1c-mediated de novo lipogenesis. It also exerts anti-inflammatory effects by inhibiting histone deacetylases (HDACs), alleviating the inflammation associated with steatohepatitis [71]. Comparable benefits have been observed in other teleosts, where butyrate enhances intestinal barrier integrity [73]. In the present study, OA-mediated restoration of butyrate levels (Figure 9) likely represents a key conduit within the gut–liver axis, supporting coordinated regulation of lipid metabolism and suppression of inflammation in Nile tilapia.

## 5. Conclusions and Research Limitations

In summary, OA functions as a multi-target modulator, not only reshaping the gut microbiome’s composition but also reprogramming its functional output. By potentially engaging the NO pathway, bile acid signaling, and butyrate-mediated crosstalk, OA reprograms hepatic cytokine expression and key metabolic regulators, thereby mitigating HFD-induced disorders in Nile tilapia.

Nevertheless, certain limitations remain. First, microbial functions were inferred using FAPROTAX and PICRUSt2, and direct validation through targeted metabolomics (e.g., bile acids and NO signaling) is necessary to confirm these pathways. Second, the observed correlations between microbiota and host gene expression do not establish causality. Future studies employing fecal microbiota transplantation or molecular agonists/antagonists are required to definitively elucidate the causal mechanisms underlying the OA-modulated gut–liver axis.

## Figures and Tables

**Table 1:** Details of representative DEGs in the PPAR, MAPK, mTOR, and autophagy–lysosome pathways.

Gene Name	Description	HFD vs. ND		OAL vs. HFD		OAH vs. HFD		Pathway
		log_2_FC	*p*	log_2_FC	*p*	log_2_FC	*p*	
*cpt2*	carnitine O-palmitoyltransferase 2	−0.11	0.82	1.38	0.00	1.60	0.00	PPAR signaling pathway
*pparab*	PPAR alpha	0.89	0.00	−2.22	0.00	−1.51	0.00	
*hmgcs1*	hydroxymethylglutaryl-CoA synthase	−0.05	0.90	−1.46	0.00	−0.96	0.00	
*ENSONIG00000002143*	retinoic acid receptor RXR-gamma-B	−0.03	0.91	−1.34	0.00	−1.11	0.00	
*ppargc1a*	PPAR gamma coactivator 1-alpha	0.55	0.15	−1.01	0.01	−0.19	0.63	
*rxrb*	retinoic acid receptor RXR-beta-A	0.92	0.05	1.46	0.00	1.54	0.00	
*map2k2a*	dual specificity MAPK kinase 2	−0.24	0.63	1.63	0.00	1.79	0.00	MAPK signaling pathway
*map2k2b*	dual specificity MAPK kinase 2	−0.16	0.73	1.07	0.00	1.02	0.00	
*mapk1*	MAPK 1	0.51	0.29	1.58	0.00	1.33	0.00	
*map3k11*	MAPK kinase kinase 11	1.59	0.02	1.60	0.00	1.35	0.00	
*prkcba*	protein kinase C beta type	NA	NA	1.67	0.00	2.09	0.00	
*map3k5*	MAPK kinase kinase 5	−0.93	0.00	1.05	0.00	1.17	0.00	
*fgfr1*	fibroblast growth factor receptor 1-A	0.12	0.80	−1.12	0.01	−1.26	0.00	
*prkcbb*	protein kinase C beta type	0.67	0.28	1.99	0.00	1.40	0.00	
*prkcaa*	protein kinase C alpha type	0.13	0.86	1.82	0.00	1.71	0.00	
*mapkapk3*	MAP kinase-activated protein kinase 2	0.25	0.62	−2.58	0.00	−1.93	0.00	
*mapkapk2*	MAP kinase-activated protein kinase 2	−1.35	0.00	2.08	0.00	1.09	0.00	
*tgfb3*	transforming growth factor beta-3	−0.32	0.49	1.26	0.00	1.11	0.00	
*ENSONIG00000005515*	ribosomal protein S6 kinase beta-1	−0.42	0.31	1.18	0.00	0.96	0.02	mTOR signaling pathway
*eif4ebp1*	eukaryotic TIF4E-binding protein 1	−0.19	0.31	−0.94	0.00	−1.16	0.00	
*rps6*	40S ribosomal protein S6	0.01	0.98	−2.07	0.00	−1.65	0.00	
*rps6ka2*	ribosomal protein S6K alpha-2-like	−1.82	0.00	4.68	0.00	2.90	0.00	
*rps6ka3b*	ribosomal protein S6K alpha-3	—	—	2.46	0.00	2.01	0.00	
*lamtor3*	ragulator complex protein LAMTOR3	−0.01	0.98	−1.10	0.00	−1.38	0.00	
*lamtor4*	ragulator complex protein LAMTOR4	0.27	0.58	−1.90	0.00	−1.24	0.01	
*badb*	bcl2-associated agonist of cell death	0.14	0.75	1.09	0.00	1.00	0.01	Autophagy-Lysosome Pathway
*rab8a*	ras-related protein Rab-8A	0.34	0.50	1.91	0.00	1.30	0.00	
*lamp2*	lysosome-associated membrane glycoprotein 2	0.10	0.80	1.31	0.00	0.98	0.01	
*dapk2a*	death-associated protein kinase 3	1.53	0.02	−1.85	0.00	−3.23	0.00	
*vmp1*	vacuole membrane protein 1	0.25	0.54	−1.43	0.00	−1.32	0.00	
*ddit4*	DNA damage-inducible transcript 4 protein	0.42	0.28	−2.10	0.00	−1.88	0.00	
*lamp1b*	lysosome-associated membrane glycoprotein 1	0.32	0.56	1.44	0.00	0.90	0.03	
*ENSONIG00000043238*	DNA damage-inducible transcript 4-like protein	−0.13	0.73	−4.05	0.00	−1.16	0.00	
*scarb2c*	lysosome membrane protein 2	0.42	0.38	1.24	0.00	1.60	0.00	
*ENSONIG00000007616*	lysosome membrane protein 2	0.49	0.40	2.40	0.00	2.57	0.00	

## Data Availability

The transcriptomic datasets are archived in the NCBI Sequence Read Archive (SRA) under BioProject PRJNA1258311, and the 16S rRNA gene sequencing data are deposited under BioProject PRJNA1456299. Other datasets supporting the findings of this study are available from the corresponding author upon request.

## Supplementary Materials

The following supporting information can be downloaded at: https://www.mdpi.com/article/10.3390/microorganisms14061247/s1, Table S1: Primer details used for qPCR. Table S2: DEGs associated with lipid metabolism. Table S3: Changes in the relative abundance of taxa at the genus level.

## Abbreviations

The following abbreviations are used in this manuscript:

OA	Oleanolic acid
HFD	High-fat diet
ND	Normal diet
NAFLD	Non-alcoholic fatty liver disease
SCFAs	Short-chain fatty acids
ER	Endoplasmic reticulum
TEM	Transmission electron microscopy
H&E	Hematoxylin and eosin
ASV	Amplicon sequence variant
DEGs	Differentially expressed genes
GO	Gene Ontology
KEGG	Kyoto Encyclopedia of Genes and Genomes
PPI	Protein–protein interaction
qPCR	Quantitative polymerase chain reaction
GC-MS/MS	Gas chromatography–tandem mass spectrometry
LDA	Linear discriminant analysis
LEfSe	Linear discriminant analysis effect size
FAPROTAX	Functional Annotation of Prokaryotic Taxa
PICRUSt2	Phylogenetic Investigation of Communities by Reconstruction of Unobserved States 2
LPL	Lipoprotein lipase
ACC	Acetyl-CoA carboxylase
FAS	Fatty acid synthase
CPT	Carnitine palmitoyltransferase
HDACs	Histone deacetylases
FXR	Farnesoid X receptor
