# Supplementary material for: Oleanolic Acid Modulates the Gut–Liver Axis to Alleviate High-Fat Diet-Induced Hepatic Lipid Deposition in Nile Tilapia (Oreochromis niloticus)
Source: Microorganisms. 2026 Jun 2;14(6):1247. doi: 10.3390/microorganisms14061247 (PMC13303720; doi:10.3390/microorganisms14061247)
Supplement: Supplementary file 1 [file microorganisms-14-01247-s001.zip › Table S1.pdf]

Table S1. Primers used for qPCR.

Gene Name	Primer Name	Sequence (5' to 3')	Product Len. (bp)	Tm
gapdh	*gapdh* F	GGATACACAGAGCACCAGGTAC	136	58°C
	gapdh R	GGCAGTCACCGTTGAAGTCT		
map2k2a	*map2k2a* F	GGAGCTGGACCTGGATGAAC	149	58°C
	map2k2a R	TGTGGCGGACCTTGTTGAC		
map2k2b	*map2k2b* F	TTGGACGACCGATCTTGGATG	145	58°C
	map2k2b R	TTAGGTGGTGGCTCATTGACAA		
mapk1	*mapk1* F	CTCTGGACCTGCTGGACAAG	122	58°C
	mapk1 R	CTCGGCAACAGGCTCATCT		
pparab	*pparab* F	TCTCCGCCGACCATTCAGT	117	58°C
	pparab R	CAGCAGATAATAGCAGCCACAA		
slc27a4	*slc27a4* F	TCGTTGTGTATGGAGTGGAAGT	155	58°C
	slc27a4 R	GAGGAAGCGGAGGAAGACAG		
plin2	*plin2* F	ATCAACAACACGCCACTCAAC	100	58°C
	plin2 R	CGTAGGTGCTGCTGTCTTCA		
gck	*gck* F	AACTTCCGTGTGATGCTTGTG	182	58°C
	gck R	TTCTTGTGCTTGATGTGGTGTT		
tnf-α	*tnf-α* F	AAGCCAAGGCAGCCATCCAT	68	58°C
	tnf-α R	TTGACCATTCCTCCACTCCAGA		
il-1β	*il-1β* F	CAAGGATGACGACAAGCCAACC	149	58°C
	il-1β R	AGCGGACAGACATGAGAGTGC		
igf1	*igf1* F	TTGTCTGTGGAGAGCGAGGCTT	103	58°C
	igf1 R	CAGCTTTGGAAGCAGCACTCGT		
tgfb2	*tgfb* F	TGCTGTGTCTCCCAAGACCT	180	58°C
	tgfb R	CGGCACTTTGACGGTACGTT		
