# Supplementary material for: Oleanolic Acid Modulates the Gut–Liver Axis to Alleviate High-Fat Diet-Induced Hepatic Lipid Deposition in Nile Tilapia (Oreochromis niloticus)
Source: Microorganisms. 2026 Jun 2;14(6):1247. doi: 10.3390/microorganisms14061247 (PMC13303720; doi:10.3390/microorganisms14061247)
Supplement: Supplementary file 1 [file microorganisms-14-01247-s001.zip › Table S2.pdf]

Table S2. DEGs associated with lipid metabolism.

Gene Name	Description	Swissprot Name	HFD *vs.* ND		OAL *vs.* HFD		OAH *vs.* HFD	
			log_2FC	*P* value	log_2FC	*P* value	log_2FC	*P* value
fasn	fatty acid synthase	FAS	-1.78	0.00	-1.08	0.00	-1.60	0.00
olah	oleoyl-ACP hydrolase	SAST	-0.55	0.18	-1.10	0.01	-0.16	0.69
hsd17b8	Oreochromis niloticus hydroxysteroid 17-beta dehydrogenase 8	DHB8	0.14	0.66	-1.12	0.00	-1.03	0.01
acaca	acetyl-CoA carboxylase	ACACA	-0.07	0.84	-2.02	0.00	-1.75	0.00
CEL	bile salt-activated lipase	CEL	1.48	0.00	1.82	0.00	2.21	0.00
CEL	carboxyl ester lipase	CEL	1.38	0.00	1.30	0.00	2.20	0.00
CEL	carboxyl ester lipase	CEL	0.38	0.18	1.27	0.00	2.12	0.00
ENSONIG00000002328	lipoprotein lipase	LIPL	-1.30	0.00	1.06	0.00	-0.44	0.18
lpl	Oreochromis niloticus lipoprotein lipase	LIPL	-0.57	0.01	0.06	0.76	-1.02	0.00
apoa4a	apolipoprotein A-I	APOA1	-5.85	0.00	NA	NA	2.67	0.00
apoa4a	apolipoprotein A-IV a	APOA1	-2.58	0.00	1.14	0.04	1.45	0.01
ENSONIG00000014666	--	APOB	1.55	0.00	2.64	0.00	2.21	0.00
ENSONIG00000040595	--	APOB	1.26	0.00	1.85	0.00	1.71	0.00
ENSONIG00000027502	--	APOC1	0.01	0.98	-1.05	0.00	-0.88	0.00
ENSONIG00000008380	very long-chain acyl-CoA synthetase	S27A2	0.15	0.74	1.10	0.00	1.39	0.00
acss2	acyl-CoA synthetase short chain family member 2	ACSA	0.39	0.34	0.29	0.42	1.03	0.01
acss2l	acyl-CoA synthetase short chain family member 2 like	ACSA	0.00	0.99	-1.96	0.00	-1.15	0.00
cpt2	carnitine palmitoyltransferase 2	CPT2	-0.11	0.82	1.38	0.00	1.60	0.00
cpt1b	carnitine palmitoyltransferase 1B (muscle)	CPT1A	-0.96	0.01	-0.17	0.67	2.03	0.00

crata	carnitine O-acetyltransferase	CACP	-0.37	0.12	1.37	0.00	1.36	0.00
cratb	carnitine O-acetyltransferase	CACP	2.03	0.00	-2.47	0.00	-2.60	0.00
ehhadh	enoyl-CoA hydratase and 3-hydroxyacyl CoA dehydrogenase	ECHP	0.43	0.23	0.86	0.01	1.24	0.00
auh	AU RNA binding protein/enoyl-CoA hydratase	AUHM	0.03	0.95	1.06	0.00	1.16	0.00
soat1	sterol O-acyltransferase 1	SOAT1	-0.47	0.11	-0.24	0.35	-1.70	0.00
soat2	sterol O-acyltransferase 2	SOAT1	0.63	0.19	2.81	0.00	3.46	0.00
gck	glucokinase	HXK4	2.42	0.00	-5.82	0.00	-3.01	0.00
