# Supplementary material for: Oleanolic Acid Modulates the Gut–Liver Axis to Alleviate High-Fat Diet-Induced Hepatic Lipid Deposition in Nile Tilapia (Oreochromis niloticus)
Source: Microorganisms. 2026 Jun 2;14(6):1247. doi: 10.3390/microorganisms14061247 (PMC13303720; doi:10.3390/microorganisms14061247)
Supplement: Supplementary file 1 [file microorganisms-14-01247-s001.zip › Table S3.pdf]

Table S3. Changes in the relative abundance of taxa at the genus level.

Taxonomy	Relative abundance				HFD vs. ND		OAL vs. ND		OAH vs. ND	
	ND	HFD	OAL	OAH	Fold Change	*P*	Fold Change	*P*	Fold Change	*P*
Mycobacterium	0.002	0.025	0.014	0.037	10.217	0.012	0.574	0.119	1.500	0.265
Clavibacter	0.059	0.000	0.001	0.007	0.000	0.007	>1000	0.040	>1000	0.005
Neochlamydia	0.001	0.022	0.016	0.066	16.248	0.015	0.730	0.440	3.013	0.026
unidentified_Pirellulaceae	0.010	0.000	0.007	0.003	0.015	0.044	48.299	0.097	20.665	0.015
Bosea	0.007	0.001	0.025	0.084	0.092	0.034	38.199	0.037	128.219	0.003
Kaistia	0.000	0.007	0.000	0.001	19.405	0.024	0.055	0.025	0.186	0.038
Alsobacter	0.008	0.000	0.000	0.001	0.048	0.007	0.096	0.171	1.837	0.523
Nordella	0.461	0.725	0.242	0.255	1.574	0.009	0.334	0.000	0.352	0.000
Gemmobacter	0.004	0.000	0.001	0.001	0.022	0.004	9.532	0.152	9.934	0.146
Plesiomonas	0.246	0.002	0.031	0.030	0.007	0.003	19.550	0.060	18.644	0.252
Legionella	0.001	0.024	0.010	0.028	15.836	0.001	0.424	0.029	1.185	0.388
Aquicella	0.002	0.001	0.000	0.000	0.245	0.013	0.020	0.132	0.000	0.126
Bdellovibrio	0.003	0.001	0.002	0.007	0.208	0.030	2.842	0.294	9.659	0.034
Phreatobacter	0.006	0.005	0.014	0.058	0.835	0.619	2.674	0.037	10.636	0.000
Terrimicrobium	0.000	0.000	0.100	0.000	3.952	0.505	>1000	0.017	0.000	0.374
unidentified_Isosphaeraceae	0.000	0.001	0.003	0.002	1.483	0.483	4.580	0.138	3.137	0.037
